# Semen sEV tRF-Based Models Increase Non-Invasive Prediction Accuracy of Clinically Significant Prostate Cancer among Patients with Moderately Altered PSA Levels

**DOI:** 10.3390/ijms251810122

**Published:** 2024-09-20

**Authors:** Adriana Ferre-Giraldo, Manel Castells, José Francisco Sánchez-Herrero, Olga López-Rodrigo, Maurizio de Rocco-Ponce, Lluís Bassas, Francesc Vigués, Lauro Sumoy, Sara Larriba

**Affiliations:** 1 Human Molecular Genetics Group-Bellvitge Biomedical Research Institute (IDIBELL), 08908 Hospitalet de Llobregat, Barcelona, Spain; aferre@idibell.cat; 2 Urology Service, Bellvitge University Hospital-ICS (Institut Català de la Salut), 08908 Hospitalet de Llobregat, Barcelona, Spain; mcastells@bellvitgehospital.cat (M.C.); fvigues@bellvitgehospital.cat (F.V.); 3 High Content Genomics and Bioinformatics (HCGB)-Germans Trias i Pujol Research Institute (IGTP), 08916 Badalona, Spain; jsanchez@igtp.cat (J.F.S.-H.); lsumoy@igtp.cat (L.S.); 4 Laboratory of Andrology and Sperm Bank, Andrology Service-Puigvert Foundation, 08025 Barcelona, Spain; olopez@fundacio-puigvert.es (O.L.-R.); mderocco@fundacio-puigvert.es (M.d.R.-P.); lbassas@fundacio-puigvert.es (L.B.)

**Keywords:** prostate cancer, tsRNAs, 5′tRFs, PSA, biomarkers, non-invasive diagnosis/prognosis, semen extracellular vesicles

## Abstract

PSA screening has led to an over-diagnosis of prostate cancer (PCa) and unnecessary biopsies of benign conditions due to its low cancer specificity. Consequently, more accurate, preferentially non-invasive, tests are needed. We aim to evaluate the potential of semen sEV (small extracellular vesicles) tsRNAs (tRNA-derived small RNAs) as PCa indicators. Initially, following a literature review in the OncotRF database and high-throughput small RNA-sequencing studies in PCa tissue together with the sncRNA profile in semen sEVs, we selected four candidate 5′tRF tsRNAs for validation as PCa biomarkers. RT-qPCR analysis in semen sEVs from men with moderately elevated serum PSA levels successfully shows that the differential expression of the four tRFs between PCa and healthy control groups can be detected in a non-invasive manner. The combined model incorporating PSA and specific tRFs (5′-tRNA-Glu-TTC-9-1_L30 and 5′-tRNA-Val-CAC-3-1_L30) achieved high predictive accuracy in identifying samples with a Gleason score ≥ 7 and staging disease beyond IIA, supporting that the 5′tRF fingerprint in semen sEV can improve the PSA predictive value to discriminate between malignant and indolent prostate conditions. The in silico study allowed us to map target genes for the four 5′tRFs possibly involved in PCa. Our findings highlight the synergistic use of multiple biomarkers as an efficient approach to improve PCa screening and prognosis.

## 1. Introduction

Male reproductive system diseases affect the health and quality of life of many men. Specifically, one of every six individuals will develop prostate cancer (PCa) during his lifetime [1]. Therefore, PCa constitutes the second most frequent cancer in men. Currently, the screening of PCa is first based on physical examination of the prostate gland (digital rectal examination—DRE) together with the determination of PSA (prostate-specific antigen) in blood, which has allowed for better detection of this malignant disease and contributed to reducing mortality. Suspicious results are then evaluated in transrectal or transperineal biopsies, essential to confirm the diagnosis. However, evidence suggests that PSA is not a true diagnostic test for PCa [2] due to the low cancer specificity that PSA presents [3], being unable to differentiate PCa from benign prostatic hyperplasia (BPH). This results in a high number of false positives of PCa and thus in a high number of unnecessary biopsies of benign disease. Specifically, the detection rate is really deficient (20% or less) in patients with PSA levels of 4 to 10 ng/mL. Thus, this PSA range is considered “a diagnostic grey zone”, where additional biomarkers for PCa are highly required to evidence the presence of a malignant disease. Additionally, there are still no reliable tests for the severity and the likely course of PCa pathology, and prognosis prediction is mostly based on the practice of tissue biopsies, which, due to the multifocal nature of prostatic carcinogenesis, are often not informative. The identification of precise and preferably non-invasive diagnostic/prognostic biomarkers for PCa is essential, which will influence the PCa treatment decisions, resulting in a more appropriate and/or personalized treatment of the patient at earlier stages, contributing to improvements in patient survival rates.

Extracellular release of molecules has been demonstrated to be related to cellular pathophysiology, which highlights the potential to detect disease-associated cellular changes by measuring molecules in body fluids as a “liquid biopsy”. In this context, semen represents an ideal biological source of biomarkers for disorders affecting organs of the male reproductive system, all of which contribute to the production of semen. Specifically, 40% of semen comes from secretions from the prostate. Thus, its contents should contain prostate-specific molecules related to the disease which can potentially be used as PCa-specific biomarkers [4,5,6].

Many of the extracellular molecules are released within membranous structures, known as extracellular vesicles (EVs), which protect their content from degradation. Both healthy and pathological cells expel EVs into the fluids. Among them, small EVs (sEVs), smaller than 200 nm in diameter which include both microvesicles and exosomes, mediate paracrine signalling through the bloodstream, lymph, or other fluids such as semen with important implications for biology, diseases, and medicine [7]. These vesicles regulate cellular behaviour, after their binding and fusion with the membrane of the target cell, and releasing the cargo they transport.

Specifically, seminal plasma contains a unique concentration of sEVs [8] secreted from the different organs of the male reproductive system, which can be transferred to recipient cells, contributing to sperm quality and the fertilization process. The RNA content of sEV in semen is enriched in small non-coding RNA (sncRNA) molecules such as microRNA (miRNA) and tRNA-derived small RNA (tsRNA) species [8,9] involved in sperm maturation and function. Both miRNAs and tsRNAs are involved in RNA silencing and post-transcriptional regulation of gene expression and thus are implicated in many physiological/pathological functions. The sEV miRNA profile in semen from PCa patients and their capacity as biomarkers have been investigated [10]; however, the potential of using semen sEV tsRNAs as PCa indicators is yet unexplored. Interestingly, epididymis and prostate are the major producers of sEV tsRNAs in the male reproductive tract (compared with the testis [11]); thus, alterations in these sEV sncRNA profiles from semen would be expected to reveal the presence of epididymal and prostate disorders and reflect their severity.

tsRNAs were first found in the urine of cancer patients [12]. Although they were initially considered as random degradation products during biogenesis, they are now known to be produced by specific nucleases and to be closely related to metabolism, viral infection, neurodegeneration, and tumorigenesis [13,14,15,16,17]. tsRNAs have critical roles in the development of most malignancies, regulating proliferation, apoptosis, migration, cancer stem cell phenotypes, and other cancer features. These sncRNAs are thought to inhibit gene expression through miRNA-like silencing, but also to modulate translation (reviewed in [18]). tsRNAs can be classified into six groups according to the region on mature/pre-tRNAs from which they are derived: 5′-tRNA halves, 3′-tRNA halves (both tsRNAs of length 30–50 nt), 5′-tRNA-derived RNA fragment (5′-tRFs), 3′-tRFs, internal tRFs (i-tRFs), and tRF-1 (or 3′U tRF), the latter four tsRNAs of 16–30 nt in length. Specifically, hundreds of tRFs, especially 5′tRFs, were found to be abnormally expressed in PCa tissue compared with normal prostate tissue [19,20,21,22,23], suggesting they have great potential as new biomarkers for cancer diagnosis/prognosis.

Studying the expression profile in semen sEV of dysregulated tRFs in PCa tissue would allow us to select candidate biomarkers for non-invasive detection with diagnostic/prognostic purposes which would help to identify PCa cases, especially those who need treatment. Special care for tRF selection should be taken as, similarly to isomiRs, individual tRNA genes can be cleaved at specific sites to produce many different types and lengths of tsRNAs that share a major part of the sequence. To assess the clinical significance of tRF biomarkers for PCa, the present study selected common 5′tRFs differentially expressed in PCa tissue from different high throughput small RNA studies [19,20,22,23] and validated their use in semen samples from men with moderately altered serum PSA levels, where the identification of truly non-invasive biomarkers of PCa is more necessary.

## 2. Results

### 2.1. Selection of Candidate tRFs Differentially Expressed in PCa Tissue to Study in Semen sEVs

Several previous studies assessed sncRNA levels in PCa tissular samples compared with the adjacent non-tumoral tissue. 5′tRF profile in PCa tissue was described to be altered in three different publications using high-throughput small RNA sequencing [19,20,22] and in the OncotRF Database (http://bioinformatics.zju.edu.cn/OncotRF/index.html (accessed on 20 September 2023)) [23].

Additionally, recent research from our group evidenced that tsRNAs together with miRNAs represented the most abundant forms of small RNAs in semen sEV: 34.14% of the human-classified small RNAs mapped to tsRNAs and 59.59% to miRNAs [9]. As the epididymis and the prostate are the major producers of sEV tsRNAs in the male reproductive tract, we suggest that some of these 5′tRFs associated with the presence of malignant cells in prostatic tissue can be detected in semen sEVs with the potential to be used as non-invasive biomarkers of PCa.

**Figure 1:**
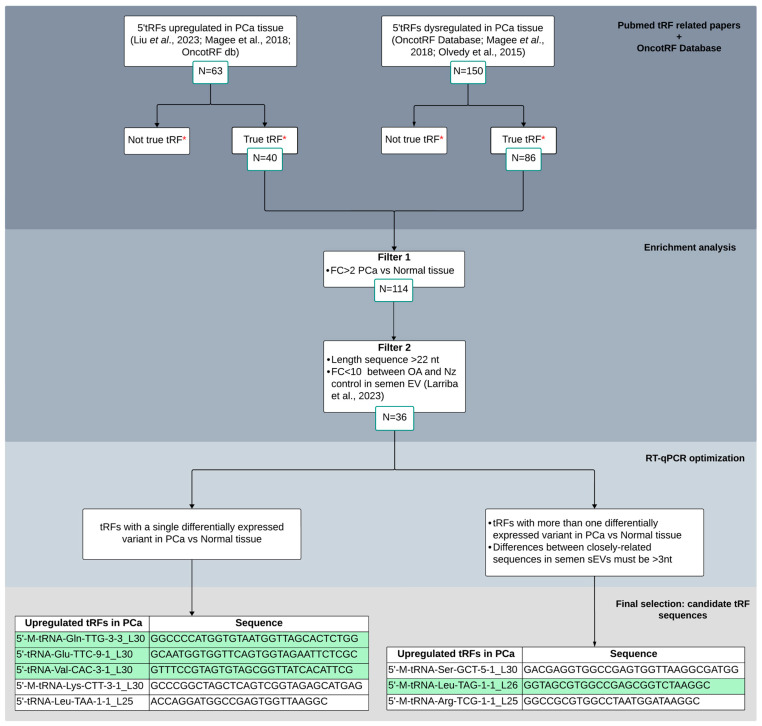
Bibliometric tissue-PCa tRF screening flowchart for candidate selection and validation as biomarkers in semen sEVs. Different criteria were considered for enrichment analysis and RT-qPCR optimization. Four tRFs that resulted in a single peak from the melting curve analysis in RT-qPCR experiments, depicted in green, were finally selected for semen validation. *: OncotRF database (http://bioinformatics.zju.edu.cn/OncotRF; accessed on 20 September 2023); OA: male infertility due to obstructive azoospermia; Nz: fertile men with normozoospermia. Larriba et al., 2020 [9]; Olvedy et al., 2015 [19]; Magee et al., 2018 [20]; Liu et al., 2023 [22].

To select candidate tRFs for validation in sEV semen samples as biomarkers for PCa, we followed a protocol/workflow based on literature and database search and additional recent research from our group (Figure 1). Initially, we selected 213 5′tRFs that appeared dysregulated in PCa tissue in two or more of the aforementioned publications. Subsequently, we discarded those 5′tRFs which were mentioned not to be true tRF in OncotRF database. As a result, we retained 126 5′tRFs to be considered for subsequent analysis.

Next, different criteria were proposed in order to select candidate 5′tRFs as potential biomarkers of PCa in semen. Common differentially expressed 5′tRFs in PCa tissue (FC > 2; *q*-value < 0.05) were first selected from the three aforementioned studies (number of counts > 10 and length > 22 nt, as PCa tissue was described to be enriched from 5′tRFs larger than 20 nt [20]). These tRFs were then tested to be present in semen sEVs (from our previous paper [9]) and those variants that do have similar expression values between vasectomized and not vasectomized healthy control individuals were selected, which resulted in 36 5′tRFs to be potentially quantified by RT-qPCR in sEV from semen.

Similarly, as it occurs with miRNAs, individual tRFs showed heterogeneity in length and/or sequence, resulting in the simultaneous presence of multiple naturally occurring variants [9], which can introduce errors in RT-qPCR measurement due to cross-reaction among near-identical variants (differing by 3 or less nt). Thus, taking into account this feature, we first selected 5′tRFs that show only one dysregulated variant in PCa compared with normal tissue. Additionally, among the differentially expressed tRFs that present closely related sequences in PCa tissue, sharing high homology of sequence, those showing differences of >3 nt in semen sEVs [9] were also selected (n = 3) for further analysis. In total, eight tRFs were finally chosen for validation in semen sEV samples (Figure 1). Interestingly, these tRFs showed relevant increased levels (FC > 9) in PCa tissue [19,20,22].

### 2.2. Clinical Assessment of the Individuals Included in the Study

**Figure 2:**
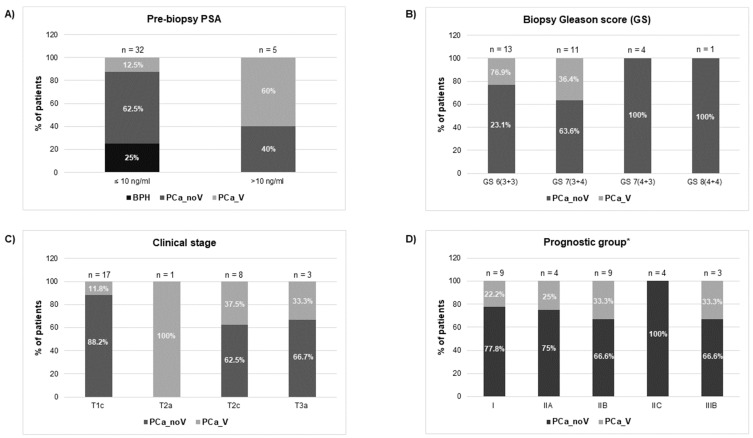
Clinical features [(**A**) prebiopsy PSA; (**B**) biopsy Gleason Score; (**C**) clinical stage; (**D**) prognostic group] of individuals who underwent prostate screening for PCa diagnosis. BPH: benign prostate hyperplasia group; PCa_noV: prostate cancer from non-vasectomized individuals; PCa_V: prostate cancer from vasectomized individuals. * American Joint Committee on Cancer Prognostic Stage grouping (8th edition).

Clinicopathological characteristics of patients and control individuals are shown in Figure 2 and Supplementary Table S1. In relation to PSA levels, most PCa cases and benign prostatic hyperplasia (BPH) controls (32 out of 37 individuals 86.5%) fail within the “grey zone” of PSA diagnostic value (4–10 ng/mL); only 5 individuals showed slightly higher PSA values (11.9–17.7 ng/mL). Both groups, BPH and PCa, were similar regarding age (mean value: 58.5).

The histopathological evaluation of prostate biopsy showed that 44.8% of PCa patients (13 out of 29 individuals) presented low-grade cancer (Gleason score GS 6) and 51.7% (fifteen samples) showed intermediate-grade cancer (GS 7), being 11 of them GS 7 (3 + 4). After the clinical staging of the tumours, 17 samples were classified as intermediate-risk tumours whereas only three samples evidenced tumour extension through the prostate capsule (stage T3a) suggesting a worse prognosis. Additionally, our samples were staged into prognostic groups (I, IIA, IIB, IIC, IIIB) in accordance with the AJCC (American Joint Committee on Cancer) PCa staging system (8th edition), which adds pre-treatment PSA and tumour Gleason grade to tumour-node-metastasis (TNM) classification [24]. Considering only the PCa samples under this prognostic classification for the analysis, a considerable number of the samples are categorised as low-/intermediate- risk tumours: [31% correspond to stage I, 13.8% stage IIA, 31% stage IIB (3 individuals of this group of patients presented PSA 10–17 ng/mL when diagnosed)], whereas near a quarter of the samples are classified as high-risk tumours: 13.8% stage intermediate-high risk IIC and 10.3% stage high risk-advanced IIIB [two of the individuals presented PSA > 10 ng/mL (12.5 ng/mL and 17.7 ng/mL) at the time of diagnosis]. It is worth noting that the different prognostic connotations between Gleason 7 (3 + 4) and (4 + 3) are reflected in this AJCC staging system: organ confined GS7 (3 + 4) samples are included in IIB group whereas organ confined GS7 (4 + 3) samples are included in IIC prognostic group.

### 2.3. PCa-Associated tRFs Show Altered Levels in Semen sEVs from Men with Prostate Carcinogenesis

We first designed miRPrimer2 primers to quantify the expression levels of the eight tRFs that were initially preselected. Only four of them resulted in a single peak from the melting curve analysis (Supplementary Figure S1), suggesting the amplification of a single tRF variant in semen sEVs. Alternative primers were designed for those tRFs that resulted in several peaks in the melting analysis without improvement. Designed primers chosen for tRF quantification by poly(A) based miRprimer2 RT-qPCR strategy of the four finally selected tRFs are described in Table 1.

**Figure 3:**
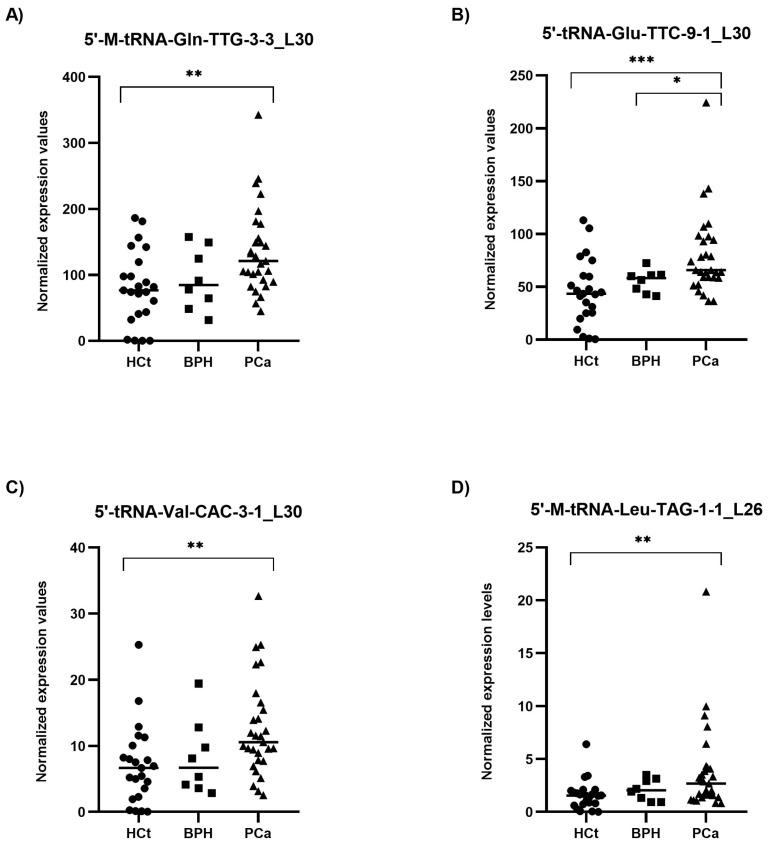
Semen sEV tRF isoform levels in benign prostate hyperplasia (BPH) and malignant prostate tumour (PCa) compared with healthy controls (HCt). Expression profiling of 5′-M-tRNA-Gln-TTG-3-3_L30 (**A**); 5′-tRNA-Glu-TTC-9-1_L30 (**B**); 5′-tRNA-Val-CAC-3-1_L30 (**C**) and 5′-M-tRNA-Leu-TAG-1-1_L26 (**D**) tRF isoforms in seminal small extracellular vesicles (sEVs), obtained by miRPrimer2 reverse transcriptase-quantitative real-time polymerase chain reaction (RT-qPCR) quantification. Data are shown as relative quantification (RQ) values, which were calculated using the 2dCq strategy and relative to the expression values of miR-30e-3p value. The horizontal bar displays the median expression value. Significant differences between groups are indicated: * *p* < 0.05; ** *p* < 0.01, *** *p* < 0.001 (Mann–Whitney U-test).

RT-qPCR results on semen sEV samples showed that expression values of the four tRFs tested: 5′-M-tRNA-Gln-TTG-3-3_L30 (*p* = 0.002), 5′-tRNA-Glu-TTC-9-1_L30 (*p* = 0.001), 5′-tRNA-Val-CAC-3-1_L30 (*p* = 0.003) and 5′-M-tRNA-Leu-TAG-1-1_L26 (*p* = 0.003), were significantly overexpressed in PCa when compared with HCt group (Figure 3). No difference in expression for any of the tRFs tested was found when comparing HCt and BPH, and, similarly to PSA, between BPH and PCa groups, except for 5′-tRNA-Glu-TTC-9-1_L30 (*p* = 0.043). There are no significant differences between vasectomised and non-vasectomised individuals in both HCt and PCa groups for all tRFs studied (Supplementary Figure S2), corroborating that none of these tRFs originates primarily from testis and/or epididymis.

Interestingly, the expression values of the four tRFs in semen sEV samples provided good and statistically significant predictive accuracy to discriminate between the presence of a malignant tumour in the prostate (PCa group) and the absence of a tumour (HCt + BPH group): 5′-M-tRNA-Gln-TTG-3-3_L30 (AUC: 0.736; *p* = 0.002), 5′-tRNA-Glu-TTC-9-1_L30 (AUC: 0.766; *p* = 0.000*), 5′-tRNA-Val-CAC-3-1_L30 (AUC = 0.727; *p* = 0.003) and 5′-M-tRNA-Leu-TAG-1-1_L26 (AUC = 0.711; *p* = 0.005) (Table 2A). To determine if a multiplex model could improve performance over single biomarkers for discriminating PCa from non-malignant samples, the four tRFs were analysed in a multivariate logistic regression analysis, resulting in a model that only includes 5′-tRNA-Glu-TTC-9-1_L30 for HCt+BPH vs PCa differentiation (Sp: 80.6%; Sn: 65.5%) (Table 2A) and for BPH vs PCa differentiation (Sp: 0%; Sn: 93.1%) (Table 2B). In the case of BPH vs PCa discrimination when tRF variables but also PSA values were included in the multivariate analysis, it resulted in a model comprising PSA + 5′-M-tRNA-Gln-TTG-3-3_L30 + 5′-tRNA-Glu-TTC-9-1_L30 with better specificity (Sp: 25%; Sn: 96.6%), high prediction accuracy (AUC: 0.759; *p*-value= 0.027) and much more useful than PSA or individual tRF for diagnosis (Table 2B).

### 2.4. 5′tRF Levels in Semen sEVs Are Associated with PCa Clinical Risk/Severity

**Figure 4:**
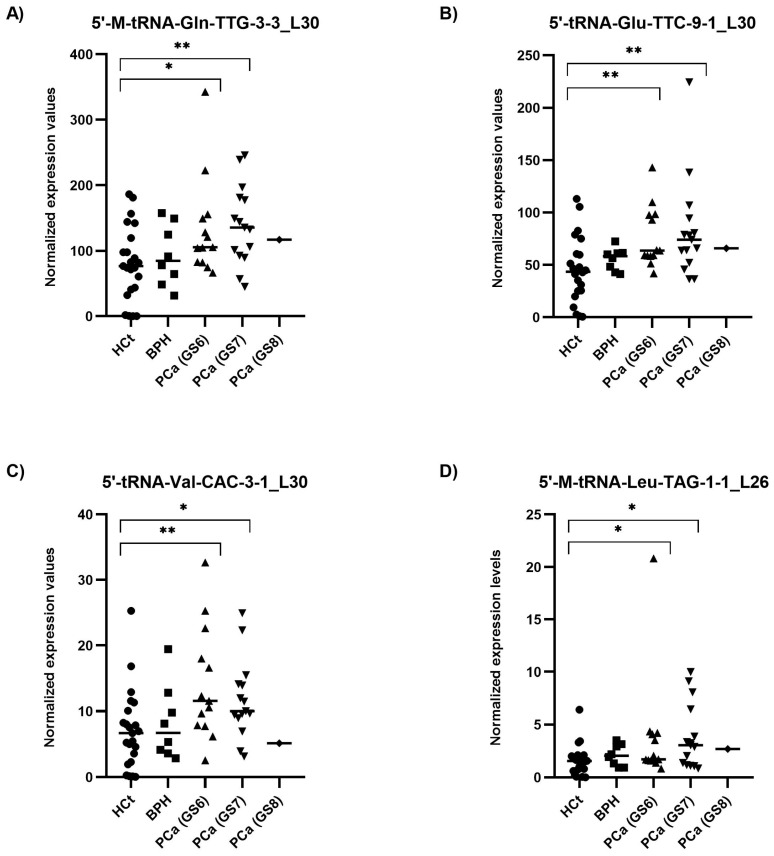
Semen sEV tRF isoform levels in PCa samples with different severities of tumour defined by Gleason Score. Expression profiling of 5′-M-tRNA-Gln-TTG-3-3_L30 (**A**); 5′-tRNA-Glu-TTC-9-1_L30 (**B**); 5′-tRNA-Val-CAC-3-1_L30 (**C**) and 5′-M-tRNA-Leu-TAG-1-1_L26 (**D**) tRF isoforms in seminal small extracellular vesicles (sEVs), obtained by miRPrimer2 reverse transcriptase-quantitative real-time polymerase chain reaction (RT-qPCR) quantification. Data are shown as relative quantification (RQ) values, which were calculated using the 2dCq strategy and relative to the expression values of miR-30e-3p value. The horizontal bar displays the median expression value. Significant differences between groups are indicated: * *p* < 0.05; ** *p* < 0.01 (Mann–Whitney U-test). HCt: healthy controls; BPH: benign prostatic hyperplasia; PCa (GS6): Gleason 6 (the least-aggressive) classified prostate cancer; PCa (GS7): Gleason 7 (a medium-grade) classified prostate cancer; PCa (GS8): Gleason 8 (aggressive) classified prostate cancer.

When the PCa samples were classified by their severity or degree of PCa affectation according to the biopsy Gleason score (GS), all four tRFs tested showed statistically significant differences between HCt and GS6 or GS7 PCa groups (Figure 4). Specifically, the expression values of three tRFs in semen sEVs resulted in good predictive accuracy to discriminate men without PCa (HCt + BPH) or with GS6 from PCa GS ≥ 7 (Table 2C): [5′-M-tRNA-Gln-TTG-3-3_L30 (AUC: 0.7 *p* = 0.018; Sn: 12.5%, Sp: 95.5%); 5′-tRNA-Glu-TTC-9-1_L30 (AUC: 0.698 *p* = 0.020; Sn: 12.5%, Sp: 97.7%), and 5′-M-tRNA-Leu-TAG-1-1_L26 (AUC: 0.666 *p* = 0.05).

Again, when a multivariate logistic regression analysis was performed, the resulting model only included 5′-tRNA-Glu-TTC-9-1_L30. Additionally we performed the same analysis considering only the individuals with prostate disease condition (excluding the HCt group) (Table 2D): similarly to PSA, no individual tRF was able to discriminate between BPH and PCa GS6 or PCa GS ≥ 7 samples, however the model obtained after multivariate logistic regression analysis including PSA and the four tRFs resulted in a good predictive accuracy (AUC: 0.780; *p* = 0.004; Sn: 85.7%; Sp: 50%), supporting that 5′tRF fingerprint in semen sEVs can improve the predictive value of PSA to discriminate individuals with malignant from indolent disease of prostate in those individuals with moderately altered PSA levels.

**Figure 5:**
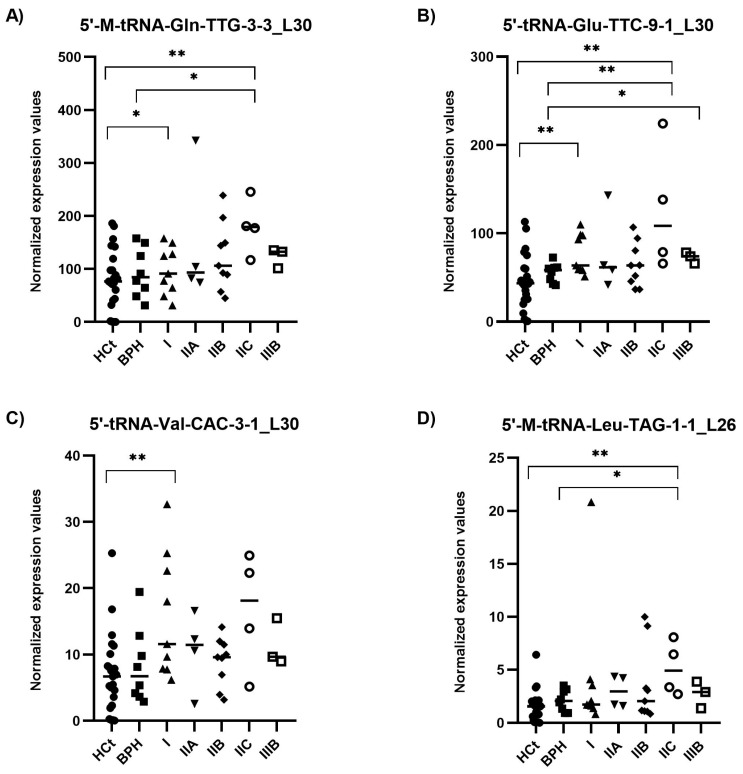
Semen sEV tRF isoform levels in clinically staged PCa samples by prognostic groups. Expression profiling of 5′-M-tRNA-Gln-TTG-3-3_L30 (**A**); 5′-tRNA-Glu-TTC-9-1_L30 (**B**); 5′-tRNA-Val-CAC-3-1_L30 (**C**) and 5′-M-tRNA-Leu-TAG-1-1_L26 (**D**) tRF isoforms in seminal small extracellular vesicles (sEVs), obtained by miRPrimer2 reverse transcriptase-quantitative real-time polymerase chain reaction (RT-qPCR) quantification. Data are shown as relative quantification (RQ) values, which were calculated using the 2dCq strategy and relative to the expression values of miR-30e-3p value. The horizontal bar displays the median expression value. Significant differences between groups are indicated: * *p* < 0.05; ** *p* < 0.01 (Mann–Whitney U-test). HCt: healthy controls; BPH: benign prostatic hyperplasia; PCa samples were staged into prognostic groups in accordance with the 8th Edition of AJCC (American Joint Committee on Cancer) staging system for PCa: from the lowest risk (PCa I) to highest risk (PCa IIIB) tumours.

Most importantly, considering the AJCC PCa staging (which describes how far cancer has spread and therefore is associated with the likely course of the disease) (Figure 5), we found the combined model including PSA + 5′-tRNA-Glu-TTC-9-1_L30 + 5′-tRNA-Val-CAC-3-1_L30 as a useful test for classifying samples within intermediate PSA levels into a prostate disease with better (BPH + PCa_I) and worse prognosis (PCa_IIA + IIB + IIC + IIIB) (AUC: 0.756; *p*-value: 0.008) (Table 2E). The same PSA-5′tRF(Glu + Val) combined model was able to discriminate (BPH + PCa I + IIA) from (PCa IIB + IIC + IIIB) samples (AUC: 0.736; *p* = 0.032; Sn: 43.8%, Sp: 85.7%; PPV: 70%, NPV: 66.7%), as well as (PCa I + IIA) from (PCa IIB + IIC + IIIB) samples (AUC: 0.750; *p* = 0.023; Sn: 68.8%, Sp: 61.5%; PPV: 68.7%, NPV: 61.5%).

In addition, the levels of the 5′tRFs were tested in prostate cancer cell lines. 5′tRFs showed a tendency to increase their expression in androgen-sensitive LNCaP cell line compared with the RWPE1 non-carcinoma human prostate cell line, whereas similar or decreased levels were observed in metastatic androgen-insensitive DU-145 and PC3 PCa cell lines (Supplementary Figure S3).

### 2.5. Prediction of the 5′tRF Target Genes

Identifying the target genes of the four putative 5′tRF biomarkers is relevant to understand their role in the initiation and/or progression of the disease. The tRFTar web platform (including the AGO-mediated interactions between 12,100 tRFs and 5688 target genes) allowed us to generate a list of genes and candidate pathways. Among the 195 genes that are predicted to interact with 5′-M-tRNA-Leu-TAG-1-1_L26, 5 target genes (*AR*, *ERBB2*, *GSTP1*, *MAP2K1*, *MTOR*) are involved in KEGG (Kyoto Encyclopaedia of Genes and Genomes) prostate cancer signalling (Table 3A). Referring 5′-M-tRNA-Gln-TTG-3-3_L30, only two genes (*H2BC3* and *CBX5*) were obtained as potential targets, whereas no target gene was obtained when including 5′-tRNA-Glu-TTC-9-1_L30 or 5-tRNA-Val-CAC-3-1_L30 in the database (Supplementary Table S2).

**Figure 6:**
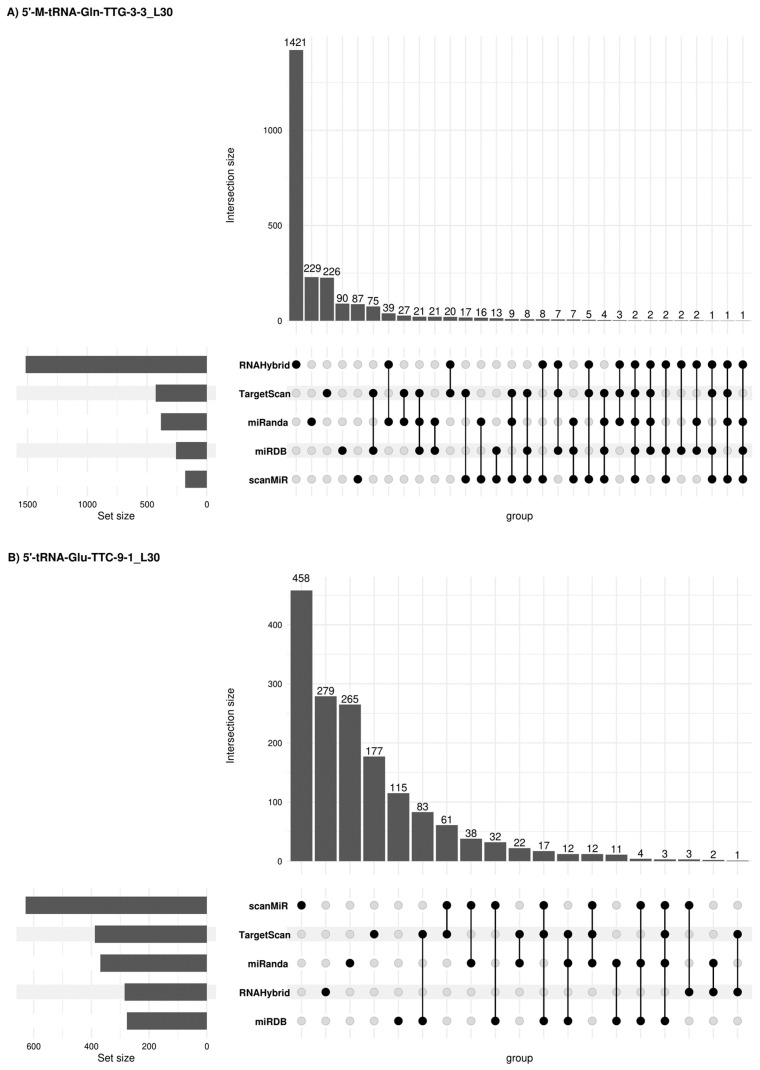

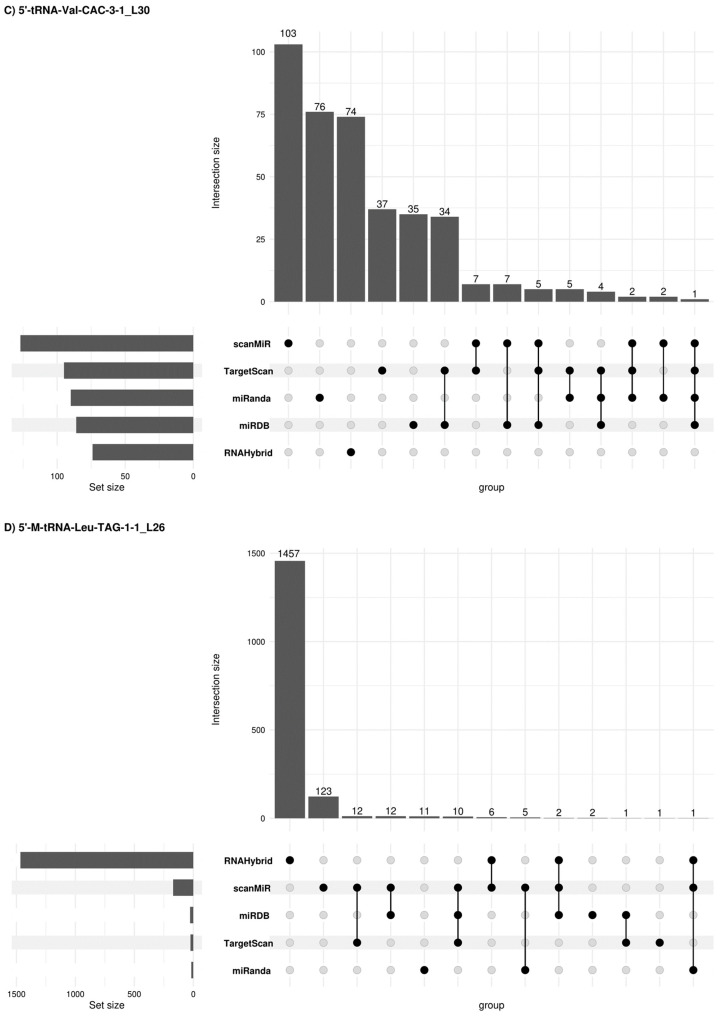
Concordant 5′tRF target genes among different prediction platforms. Upset plots [26] show all possible relationships (commonality and differences) between the elements obtained from several miRNA target gene predictive platforms for each of the four 5′tRFs tested. For each row, the cells that are part of an intersection are filled in. If there are multiple filled-in cells, they are connected with a line. An overview of the list size is also provided.

Due to potential similarities in the mechanism of expression regulation between tsRNAs and miRNAs, other alternative miRNA target gene predictive platforms were used such as sRNAtools (which uses miRanda and RNA Hybrid tools), TargetScan, miRDB (inclusion criteria: Target score > 50) and scanMiR (in this case, Kd < −1 was used as inclusion criteria) algorithms which allowed us to generate a list of target genes and candidate pathways for the 5′tRFs in semen sEVs (Figure 6). A list of 2366, 1595, 392 and 1643 target genes was obtained when using these platforms for 5′-M-tRNA-Gln-TTG-3-3_L30, 5′-tRNA-Glu-TTC-9-1_L30, 5′-tRNA-Val-CAC-3-1_L30 and 5′-M-tRNA-Leu-TAG-1-1_L26 respectively. Some concordant findings were achieved among the different approaches (Supplementary Table S3). Functional enrichment analysis, when considering concordant target genes in 3 or more platforms, showed that 56 out of the 75 target genes for 5′-M-tRNA-Gln-TTG-3-3_L30 were involved in the regulation of biological process (GO: 0050789) and 35 out of 45 target genes for 5′-tRNA-Glu-TTC-9-1_L30, are located in the membrane (GO:0016020) (Supplementary Table S4).

Thirty-one deregulated target genes are involved in KEGG prostate cancer signalling (Table 3B). Interestingly, among the eleven 5′-tRNA-Glu-TTC-9-1_L30-target genes, the interaction of six of them (*E2F3*, *PTEN*, *CREB3L2*, *CREB5*, *CREB1* and *NRAS* genes) was predicted with two or three different miRNA target gene predictive platforms, supporting the veracity of the result.

## 3. Discussion

One of the most important objectives in male urology is PCa diagnosis at an early stage preferentially in a non-invasive way. To do that, PCa screening has relied on plasma PSA as a biomarker, despite presenting critical limitations in specificity, which has led to an overdiagnosis of PCa. Not only that, as PCa usually behaves as a slow-growing tumour, screening intervention for predicting the course of the disease has been proposed as a matter of consideration: PCa biomarkers that efficiently discriminate aggressive from indolent tumours (that do not require clinical intervention but should undergo active surveillance) would decrease concerns regarding a probable overtreatment of patients. Specifically, the prognosis of men with intermediate risk localized PCa is the most difficult to predict. Therefore, secondary screening tools using either more accurate biomarkers or multiple biomarker tests (that would improve the accuracy of a single biomarker) are highly required in personalized medical care.

In this context, biomarkers in semen sEVs are emerging not only for male infertility but also for other disorders that affect the male reproductive system such as prostate cancer, which can complement current diagnostic tools available in the clinics [27]. In a previous publication, we considered semen sEV miRNAs as valuable for PCa biomarker research [10]. We now open the investigation to other small non-coding RNA regulatory molecules that are found highly expressed in semen sEVs such as tsRNAs.

Recent research from our group evidenced that, while tsRNAs are the second most abundant sncRNA forms in semen sEV following miRNAs [9], tsRNAs were found to have scarce potential as biomarkers for infertility disorders. As they mainly originate from epididymis and prostate, we reasoned that they could be explored as potential semen biomarkers of PCa.

Our preliminary analysis of bibliographic results showing a common altered profile of 5′tRFs in PCa tissue [19,20,22]) was the starting point for selecting candidate 5′tRFs for validation as PCa biomarkers in semen sEVs. Their subsequent analysis in semen sEV samples from men with moderately altered serum PSA levels, allowed us to successfully recognize that the differential expression of the four 5′tRFs in PCa when compared with healthy control individuals can be also detected in a non-invasive manner. This replication provides evidence that these tRF sequences individually or in combination should be considered as reliable PCa biomarkers in semen sEV to be further investigated. tsRNAs may derive from either nuclear or mitochondrial-encoded tRNA sequences [28]. Two of the semen sEV tRF candidate biomarkers are of mitochondrial and the other two of nuclear origin supporting a potential mitochondria-nucleus crosstalk in PCa contributing to the final phenotype expression.

Firstly, we focussed our attention on the 5′tRF expression dysregulation in PCa when compared with healthy controls. The high expression of the four 5′tRF in BPH/PCa tissue and the corresponding semen EVs support their usefulness for clinical diagnosis, reflecting the prostate health. The expression behaviour of 5′tRF-Glu-TTC-9-1_L30 shows the most accurate correlation. In line with this result, other tRNA-Glu cleavage products have resulted to have clinical significance such as the tRF-Glu-TTC-2 (tRF-31-86V8WPMN1E840) previously described to be overexpressed in PCa, promoting PCa proliferation as an oncogene [21]. Our present data evidence that the concentration of each of the 4 tRFs tested in sEVs can individually discriminate between tumoral and non-tumoral samples, much better than PSA levels in the blood. However, the potential to distinguish between BPH and PCa samples is not so clear and only one of them (5′-tRNA-Glu-TTC-9-1_L30) was found to be differentially expressed in PCa when compared to BPH. Interestingly, adding PSA to the 5′tRF model (5′-M-tRNA-Gln-TTG-3-3_L30 +5′-tRNA-Glu-TTC-9-1_L30) increases the PSA or 5′-tRNA-Glu-TTC-9-1_L30 specificity and the true negative outcome of the predictive model, being more suitable for clinical diagnosis of patients with moderately increased levels of PSA.

Although the individuals in this study exhibit low PSA levels (considered in the diagnostic grey zone) and we would expect low-risk tumours, at least 10% of them do not meet these criteria. Current diagnosis approaches for PCa cannot reliably differentiate between fast-progressing cancer requiring accurate treatment and indolent forms of disease that can be managed with active surveillance. Again, our results suggest that 5′tRF levels in semen sEVs can improve the predictive value of PSA to discriminate indolent from aggressive disease of prostate: combined PSA-tRF model (PSA + 5′-tRNA-Glu-TTC-9-1_L30 + 5′-tRNA-Val-CAC-3-1_L30) showed a clinically useful predictive accuracy to identify a group of men with GS ≥ 7 as well as to identify > IIA prognosis disease staging. Therefore, the use of a biomarker combination panel needs to be considered, to increase diagnostic accuracy and better manage PCa protocols. In our hands, the 5′tRFs are better amplified than miRNAs in semen sEVs enabling high-throughput detection in a small amount of RNA from semen sEVs, which is advantageous for their clinical translation.

tRNA-derived small RNAs have been widely explored as potential novel diagnostic biomarkers for cancer in serum/plasma [29]. Analogously, several studies have been previously carried out to identify specific PCa RNA biomarkers in plasma and urinary EVs [30]. As the prostate is the major contributor of EVs in the ejaculate, using EVs in semen, instead of other fluids like urine, is preferable for detecting sncRNAs as biomarkers for PCa, supported by our previous and current semen EV sncRNA findings. Additionally, semen offers an inherent advantage as it serves as a comprehensive liquid biopsy of the entire prostate gland, sourced from all its sections during muscle contractions, whereas, testing for PCa biomarkers in urine collected post prostate massage may only provide insights into the health of the posterior part of the gland, potentially overlooking the overall prostate health.

The involvement of tRFs in an appropriate or impaired cellular function, leading to disease such as cancer, is mediated by the regulation of mRNA stability by conjunction with different Ago proteins or directly interacting with mRNAs. Similarly to miRNAs, 5′tRFs associated with Argonaute proteins, target a wide range of transcripts leading to gene silencing [15]. However, subsequent studies, revealed that 5′tRFs are involved in translation inhibition by AGO-dependent and AGO-independent methods, the latter requiring a conserved sequence of the tRF [31,32] but no complementary sites of target sequences, supporting their role in post-transcriptional modulation. Taking into account these considerations, several predictive platforms were used to reveal potential 5′tRF-target genes: tRFTar web platform as well as TargetScan, miRDB, miRanda, RNA Hybrid and scanMiR tools were used. Surprisingly, a scarce number of concordant findings were revealed among three or more of the different approaches.

Interestingly, 5′-tRNA-Glu-TTC-9-1_L30 interaction with PCa-involved *E2F3*, *PTEN*, *CREB3L2*, *CREB5*, *CREB1* and *NRAS* genes was predicted with several different platforms supporting data veracity. Deregulation of these genes in PCa has been associated with epigenetic changes such as in lncRNA/miRNA expression [33], among other factors. E2F3 is a transcriptional activator that is amplified or overexpressed in several tumours including those of the prostate [34]. E2F3 levels have a critical role in modifying cellular proliferation rates in human prostate cancer by removing retinoblastoma protein (pRB) suppressor control at the G1/S transition in the cell cycle. In prostate cancer E2F3 overexpression is linked to tumour aggressiveness [35]. As refers to PTEN, the loss of function of this tumour suppressor is associated with the development of castration-resistant prostate cancer (CRPC). Although the genetic mechanism(s) that results in the loss of function of PTEN during the development of CRPC are not well understood, epigenetic factors such as deregulation of small regulatory RNA miRNAs have been suggested to mediate the loss of PTEN function during PCa progression [36]. Concerning CREB3L2, it collaborates with the androgen receptor to regulate the ER-to-Golgi trafficking pathway to drive prostate cancer progression [37]. CREB5 is associated with advanced PCa and was found to be overexpressed in metastatic castration-resistant prostate cancers promoting antiandrogen therapy resistance [38]. The transcription factor CREB1 is also upregulated in PCa determining cancer-relevant cell cycle, pro-survival and metabolic gene expression patterns associated with poor clinical outcomes [39]. Regarding *NRAS*, it was previously found significantly overexpressed in PCa tissue specimens [40] and an increase in N-Ras membrane expression in the transition from hormone-sensitive to hormone-refractory prostate cancer was associated with shorter time to relapse and shorter disease-specific survival [41]. Interestingly, the N-RAS expression behaviour is in line with our tRF results in prostate cell lines which show that the transcription rate of these tRFs is increased in androgen-sensitive PCa cells. Furthermore, the observed downregulation in the androgen-insensitive PCa cell lines suggests there is a loss of association of 5′tRF levels to PCa when they acquire androgen-independent properties.

In conclusion, our results provide evidence that altered 5′tRF expression in PCa tissue can also be detected in sEVs from semen. Our proposal based on 5′tRFs in semen sEVs can improve non-invasive PCa diagnosis by discriminating between malignant and indolent disease affecting the prostate, as well as prognosis prediction of PCa disease. Although the PSA testing is not able to discriminate PCa from BPH individuals presenting PSA levels in the grey zone, combining PSA with the tRF biomarker model increased AUC to be clinically used. Thus, our results are in line with previous reports suggesting the synergistic use of multiple marker fingerprint as a more efficient approach for the detection of cancer.

## 4. Materials and Methods

### 4.1. Subjects of Study

The study participants, both patients and controls, were selected among men referred to the Urology Service of the Bellvitge Hospital and the Andrology Service of Fundació Puigvert. The study was approved by the Ethical Review Board of both institutions and all the participants signed a written informed consent. All methods were performed in accordance with the relevant guidelines and regulations.

Semen samples were obtained from 10 semen donors, 2 healthy individuals consulting for vasectomy [HCt_noV; age (years): 31.33 ± 8.31] plus 11 vasectomized men [HCt_V; age (years): 39.82 ± 9.46] all of whom will define the control group 1: HCt, and 37 individuals consulting for diagnosis of PCa who underwent routine prostate screening including PSA testing and DRE. Patients who presented moderately altered PSA levels (4–18 ng/mL) were selected with consent to undergo prostate biopsy. The latter group consisted of: 29 men with biopsy-proven PCa including men who were previously successfully vasectomised [PCa-V, n = 7; age (years): 58 ± 9.69] and non-vasectomised individuals [PCa-noV, n = 22; age (years): 59.04 ± 6.31]; and additionally, 8 non-vasectomised individuals with benign prostatic hyperplasia (BPH) or prostate enlargement [control group 2; age (years): 58.62 ± 4.50] who presented elevated PSA levels (>4 ng/mL) but no detectable cancer on biopsy (Figure 2; Supplementary Table S1). We also stratified samples by tumour class based on the 8th edition of AJCC prognostic groups [42] (which consider TNM data, pre-treatment PSA levels, and tumour Gleason grade, the latter deserving special concern) into I (low risk, including organ confined GS6 samples and PSA < 10 ng/mL), IIA (low-intermediate risk, which includes organ confined GS6 samples and PSA > 10 ng/mL), IIB (intermediate risk, including organ confined GS7 (3 + 4) samples), IIC (intermediate-high risk, which includes organ confined GS7 (4 + 3) samples), IIIB (high risk-advanced due to extra-prostatic extension of the tumour, includes cT3 samples belonging to any GS grade group and PSA levels).

Four BPH, 15 PCa-noV and 6 PCa-V samples had been also previously used [10] for molecular investigation.

### 4.2. Cell Culture and Reagents

The following androgen-insensitive PCa cell lines were used: PC3 and DU145 (kindly provided by Mireia Olivan, PhD, Research Institute of Vall Hebron Hospital, Barcelona, Spain), as well as the androgen-sensitive LNCaP cancer cell line and the RWPE1 normal prostate cell line, both provided by Álvaro Aytés, PhD (Catalan Institute of Oncology ICO-IDIBELL, Hospitalet de Llobregat, Barcelona, Spain). PC3 and DU145 were grown in RPMI-1640 + GlutaMAX (Gibco, ThermoFisher Scientific, Waltham, MA, USA) supplemented with 10% fetal bovine serum (FBS), 100 U/mL penicillin, 100 µg/mL streptomycin, MEM non-essential amino acids without L-glutamine, and sodium pyruvate 1 mM (all from Gibco). LNCaP and RWPE1 were grown in RPMI-1640 medium modified to contain 2 mM L-glutamine, 25 mM HEPES, 2000 mg/L D-glucose, and 2000 mg/L sodium bicarbonate (CULTEK S.L.U., Madrid, Spain) and 10% FBS. Cells were collected and stored at −80 °C until needed.

### 4.3. Semen Samples and sEV Isolation

Semen samples were collected by masturbation following 3–5 days of sexual abstinence. The samples were then liquefied for 30 min at 37 °C. sEVs were isolated by differential centrifugation steps (1600× *g* for 10 min, then 16,000× *g* for 10 min at 4 °C), which included one microfiltration step (0.22 μm pore size) and ultracentrifugation (100,000× *g* for 2 h at 4 °C) as previously described [43,44]. Nanoparticle tracking analysis was conducted using NanoSight NS300 (Malvern Instruments Ltd.; Malvern, UK) [10].

### 4.4. Small RNA-Containing Total RNA Isolation

To degrade the residual RNA outside the vesicles, the sEV suspension was treated with RNAse A (Qiagen NV; Hilden, Germany) (100 μg/mL final reaction concentration; 15 min at 37 °C). Total RNA extraction from sEV was performed using the miRNeasy Micro Kit (Qiagen) following the previously established protocol [8]. RNA concentration was calculated by using the QUBIT fluorometer and the Quant-iT RNA Assay kit (Invitrogen; Carlsbad, CA, USA). All RNA samples presented an OD 260/280 nm ratio ≥ 1.65 when using a Nanodrop UV-Vis spectrophotometer (ThermoFisher Scientific; Waltham, MA, USA).

### 4.5. tRF Quantification by miRPrimer2 RT-qPCR Strategy

Reverse transcription of 40 ng of semen sEV total RNA in a final volume of 10 µL in the presence of ATP, RT primer (50-CAGGTCCAGTTTTTTTTTTTTTTTVN, where V is A, C and G and N is A, C, G and T), poly(A) polymerase, dNTPs, and Superscript IV enzyme (200 U/µL) (Invitrogen, ThermoFisher Scientific) at 42 °C for 1 h as previously described [45]. We found that 5′tRF sequences are better amplified than miRNA sequences, thus cDNA samples were diluted (8×) for miRNA quantification and (40×) for tRF quantification and 10 µL PCR reactions were performed (in duplicates) with 1 µL of diluted cDNA, 5 µL of 2× SYBR Green mix (Roche; Basilea, Switzerland), and 250 nM of forward and reverse primers designed by miRprimer2 software version 2.0 (https://zenodo.org/record/1339289#.Ymj5i_exVGE (accessed on 4 October 2023)) [45]. Cycling conditions were set up as follows: 5 min at 95 °C; 40 cycles of 10 s at 95, followed by 30 s at 60 °C, and finally melting curve analysis 60 °C to 99 °C. qPCR was also performed on a LightCycler^®^ 96 Instrument (Roche). The expression value of hsa-miR-30e-3p was used for data normalization, previously described to be among the most stable assays in PCa sEV semen samples [10]. The RQ values were calculated using the 2^dCp^ strategy. Sequences of primers are detailed in Table 1, including primers for hsa-miR-30e-3p previously described [25].

The same procedure was applied to determine cell expression profiling of tsRNA candidates with some modifications: 20 ng of cellular RNA were reverse transcribed, and cDNA was diluted (8×) for miRNA quantification and (16×) for tRF quantification.

### 4.6. Determining In Silico Target Genes of tsRNAs

Identification of target genes and pathways potentially altered by the tsRNA signature was first performed using the tRFTar web-based platform (http://www.rnanut.net/tRFTar/ (accessed on 6 May 2024)) [46]. The tRFTar predicted targets were determined experimentally as part of 146 crosslinking-immunoprecipitation and high-throughput sequencing (CLIP-seq) datasets.

As some tRFs have been suggested to exert their biological functions by acting as miRNAs, additional analysis of tRF target specificity was performed by using alternative miRNA-target platforms such as sRNAtools (https://rnainformatics.org.cn/sRNAtools/ (accessed on 12 May 2024)) [47] which uses sncRNA target detection tools such as miRanda and RNAHybrid]; as well as TargetScan (https://www.targetscan.org/vert_80/ (accessed on 12 May 2024)), miRDB (https://mirdb.org/ (accessed on 12 May 2024)) algorithms and scanMiR toolkit (as described Soutschek et al. (2022) [48]). The latter is based on the approach proposed by McGeary et al. (2019) [49] which substantially improved the prediction of cellular repression, thereby providing a biochemical basis for quantitatively integrating miRNAs into gene-regulatory networks). Specifically, by using scanMiR, we can obtain for each regulatory RNA the repression score, which corresponds to a log-fold change of occupancy in experimental conditions, as a quantitative measure for any desired mRNA transcript of interest or available in the genome. The value of repression is also adjusted by 3′UTR and ORF length, and scanMiR controls for supplementary pairing alignments out of the 3′ UTR. As an example, a Kd = −1 corresponds to a level of experimental occupancy twice the background. Analysis was done at the level of transcript sequence and all available transcripts for each gene were considered and smaller repression was retained.

Some R-packages of annotation such as GenomicRanges (v1.56) [50], BioMart (v2.6) [51] or specific packages for *Homo sapiens* gene annotation [EnsDb.Hsapiens.v86 (v2.99), BSgenome.HSapiens.NCBI.GRCh38 (v1.3)], were also used.

Functional enrichment analysis for characterizing a gene list was determined by using the gProfiler2 program (v0.2.3) with g:SCS multiple testing correction method applying significance threshold of 0.05.

### 4.7. Statistical Analysis

The non-parametric Mann–Whitney U-test was used to evaluate differences in relative expression of selected 5′tRFs between groups. Multivariate binary logistic regression (backward stepwise, conditional, method) and receiver operating characteristic (ROC) curve analysis of the RQ values were used to distinguish the samples showing malignancy of the prostate tumour. Accuracy was measured as the area under the ROC curve (AUC). The threshold value was determined by Youden’s index, calculated as sensitivity plus specificity-1. All data analyses were performed using the SPSS software (version 15.0; SPSS Inc.; IBM; Chicago, IL, USA). A *p*-value ≤ 0.05 was considered significant.

### 4.8. Data Visualization

For the visualization of results and intersection of different software results, the R package ComplexUpset (v1.3.5) and ggplot2 (v.3.5) were used.

## Figures and Tables

**Table 1:** tRF sequences and miRPrimer2 sequences.

tRF	ID	RNA Sequence	Chromosome (tRNA Number)	miRPrimer2 Forward Primer	miRPrimer2 Reverse Primer
5′-M-tRNA-Gln-TTG-3-3_L30	tRF-30- -6RJ89O9NF5W8	GGCCCCAUGGUGUAAUGGUUAGCACUCUGG	6 (tRNA130); 6 (tRNA173); 6 (tRNA174)	ccccatggtgtaatggttag	cagtttttttttttttttccagagtg
5′-tRNA-Glu-TTC- -9-1_L30	tRF-30- -PER8YP9LON4V	GCAAUGGUGGUUCAGUGGUAGAAUUCUCGC	2 (tRNA17)	aatggtggttcagtggtaga	ccagtttttttttttttttgcgaga
5′-tRNA-Val-CAC- -3-1_L30	tRF-30- -79MP9PMNH5IS	GUUUCCGUAGUGUAGCGGUUAUCACAUUCG	19 (tRNA13)	cgtagtgtagcggttatcac	gtccagtttttttttttttttcgaatg
5′-M-tRNA-Leu-TAG-1-1_L26	tRF-26- -RPM830MMUKD	GGUAGCGUGGCCGAGCGGUCUAAGGC	17 (tRNA42)	gtagcgtggccgag	ccagtttttttttttttttgccttag
hsa-miR-30e-3p	tRF-30- -6RJ89O9NF5W8	CUUUCAGUCGGAUGUUUACAGC		* gcagctttcagtcggatgt	* tccagtttttttttttttttgctgt

Note. The RNA sequence that corresponds to miRPrimer2 forward primer is depicted in blue whereas that corresponding to sequence complementary to reverse primer is depicted in green. * Described in Ferre et al., 2023 [25].

**Table 2:** Receiver operating characteristic (ROC) analysis showing the predictive efficiency of 5′tRFs in seminal small extracellular vesicles for PCa diagnosis.

Markers	AUC (*p*-Value)	95% CI	Sensitivity %	Specificity %	PPV %	NPV %
**A.** (HCt + BPH) vs. PCa						
5′-M-tRNA-Gln-TTG-3-3_L30	0.736 (**0.002**)	0.610–0.862	55.2	71	64	62.8
5′-tRNA-Glu-TTC-9-1_L30	0.766 (0.000 *)	0.647–0.886	65.5	80.6	76	71.4
5′-tRNA-Val-CAC-3-1_L30	0.727 (**0.003**)	0.599–0.855	51.7	77.4	68.2	63.1
5′-M-tRNA-Leu-TAG-1-1_L26	0.711 (**0.005**)	0.582–0.840	51.7	80.6	71.4	64.1
**B.** BPH vs. PCa						
PSA	0.580 (0.495)	0.371–0.789	100	0	78.4	0
5′-M-tRNA-Gln-TTG-3-3_L30	0.694 (0.097)	0.475–0.913	100	12.5	76.3	1
5′-tRNA-Glu-TTC-9-1_L30	0.737 (**0.042**)	0.574–0.901	93.1	0	77.1	0
5′-tRNA-Val-CAC-3-1_L30	0.681 (0.121)	0.465–0.897	100	0	78.4	0
5′-M-tRNA-Leu-TAG-1-1_L26	0.625 (0.285)	0.426–0.824	100	0	78.4	0
Combined PSA-tRF model (PSA + Gln + Glu)	0.759 (**0.027**)	0.591–0.927	96.6	25	82.3	66.6
**C.** (HCt + BPH + PCa_GS6) vs. (PCa GS7 + GS8)						
5′-M-tRNA-Gln-TTG-3-3_L30	0.7 (**0.018**)	0.555–0.846	12.5	95.5	50	75
5′-tRNA-Glu-TTC-9-1_L30	0.698 (**0.020**)	0.551–0.846	12.5	97.7	67	75.5
5′-tRNA-Val-CAC-3-1_L30	0.617 (0.168)	0.468–0.767	0	100	0	73.3
5′-M-tRNA-Leu-TAG-1-1_L26	0.666 (**0.05**)	0.505–0.827	0	97.7	0	72.8
Combined tRF model (Gln + Glu + Val)	0.658 (0.064)	0.505–0.810	12.5	97.7	66.7	75.4
**D.** (BPH + PCa_GS6) vs. (PCa_GS7 + GS8)						
PSA	0.670 (0.081)	0.487–0.852	31.3	76.2	50	59.2
5′-M-tRNA-Gln-TTG-3-3_L30	0.628(0.187)	0.443–0.813	18.8	90.5	60	59.4
5′-tRNA-Glu-TTC-9-1_L30	0.616 (0.232)	0.421–0.811	18.8	90.5	60	59.4
5′-tRNA-Val-CAC-3-1_L30	0.504 (0.963)	0.316–0.693	0	100	0	56.7
5′-M-tRNA-Leu-TAG-1-1_L26	0.571 (0.462)	0.377–0.766)	6.3	95.2	50	57.1
Combined tRF model (Glu + Val)	0.673 (0.075)	0.494–0.851	37.5	85.7	55.5	60.7
Combined PSA-tRF model (PSA + Glu + Val)	0.732 (**0.017**)	0.553–0.911	43.8	85.7	70	66.6
Combined PSA-tRF model (PSA + Gln + Glu + Val+ Leu)	0.780 (**0.004**)	0.615–0.944	50	85.7	72.7	69.2
**E.** (BPH + PCa_I) vs. (PCa_IIA + IIB + IIC + IIIB)						
PSA	0.629 (0.180)	0.447–0.812	50	70.6	66.7	54.5
5′-M-tRNA-Gln-TTG-3-3_L30	0.606 (0.273)	0.422–0.789	60	47.1	57.1	50
5′-tRNA-Glu-TTC-9-1_L30	0.604 (0.279)	0.416–0.793	70	52.9	63.6	60
5′-tRNA-Val-CAC-3-1_L30	0.507 (0.939)	0.312–0.703)	90	23.5	58.1	66.7
5′-M-tRNA-Leu-TAG-1-1_L26	0.606 (0.273)	0.421–0.791	100	0	54.1	0
Combined tRF model (Glu + Val)	0.697 (**0.041**)	0.527–0.867	65	52.9	61.9	56.2
Combined PSA-tRF model (PSA + Glu + Val)	0.756 (**0.008**)	0.592–0.920	70	76.5	77.8	68.4

Note. *p*-Value < 0.05 is depicted in bold; * *p*-value < 0.0001. When the multivariate logistic regression analysis of 5′tRFs variants resulted in a model including a unique 5′tRF, those are depicted in red.

**Table 3:** Potential target genes of semen sEV dysregulated tRFs involved in prostate cancer signalling (KEGG).

tRF	Target Gene	Ensembl ID (Human Gene)	Description	Molecular Function					
***A. tRFtar: tRF-target gene interaction prediction***									
5′-M-tRNA-Leu-TAG-1-1_L26	*AR*	ENSG00000169083	androgen receptor [KO:K08557]	Steroid-hormone activated transcription factor					
	*ERBB2*	ENSG00000141736	erb-b2 receptor tyrosine kinase 2 [KO:K05083] [EC:2.7.10.1]	Bind tightly to other ligand-bound EGF receptor family members to form a heterodimer, and enhancing kinase-mediated activation of downstream signalling pathways					
	*GSTP1*	ENSG00000084207	glutathione S-transferase pi 1 [KO:K23790] [EC:2.5.1.18]	Catalyses the conjugation of many hydrophobic and electrophilic compounds with reduced glutathione					
	*MAP2K1*	ENSG00000169032	mitogen-activated protein kinase 1 [KO:K04368] [EC:2.7.12.2]	It is a mitogen-activated protein (MAP) kinase involved in many cellular processes such as proliferation, differentiation, transcription regulation and development					
	*MTOR*	ENSG00000198793	mechanistic target of rapamycin kinase [KO:K07203] [EC:2.7.11.1]	Kinase which mediates cellular responses to stresses such as DNA damage and nutrient deprivation.					
***B. miRNA-target gene prediction tools***									
					**TargetScan**	**miRDB**	**miRanda**	**RNA-Hybrid**	**scan-MiR**
5′-M-tRNA-Gln-TTG-3-3_L30 * 5′-M-tRNA-Leu-TAG-1-1_L26 ^#^	*PDPK1*	ENSG00000140992	3-phosphoinositide dependent protein kinase 1 [KO:K06276] [EC:2.7.11.1]	Involved in cell surface receptor signalling pathway; regulation of protein kinase activity; and regulation of signal transduction	Yes *			Yes ^#^	
	*IKBKG*	ENSG00000269335	inhibitor of nuclear factor kappa B kinase regulatory subunit gamma [KO:K07210]	The regulatory subunit of the inhibitor of kappaB kinase (IKK) complex, which activates NF-kappaB resulting in activation of genes involved in inflammation, immunity, cell survival, and other pathways.				Yes *^,#^	
5′-M-tRNA-Gln-TTG-3-3_L30 * 5′-tRNA-Val-CAC-3-1_L30 ^$^	*IGF1R*	ENSG00000140443	insulin like growth factor 1 receptor [KO:K05087] [EC:2.7.10.1]	This receptor binds insulin-like growth factor with a high affinity. It has tyrosine kinase activity. The IGF1R plays a critical role in transformation events.	Yes *^,$^	Yes ^$^	Yes *^,$^		
5′-tRNA-Glu-TTC-9-1_L30 ^a^ 5′-tRNA-Val-CAC-3-1_L30 ^$^	*CREB3L2*	ENSG00000182158	cAMP responsive element binding protein 3 like 2 [KO:K09048]	Transcriptional activator	Yes ^a,$^	Yes ^a,$^			Yes ^$^
5′-tRNA-Glu-TTC-9-1_L30 ^a^ 5′-M-tRNA-Leu-TAG-1-1_L26 ^#^	*BRAF*	ENSG00000157764	B-Raf proto-oncogene, serine/threonine kinase [KO:K04365] [EC:2.7.11.1]	Regulates the MAP kinase/ERK signalling pathway, which affects cell division, differentiation, and secretion				Yes ^#^	Yes ^a^
5′-M-tRNA-Gln-TTG-3-3_L30	*FOXO1*	ENSG00000150907	forkhead box O1 [KO:K07201]	Transcription factor which may play a role in myogenic growth and differentiation	Yes				
	*TMPRSS2*	ENSG00000184012	transmembrane serine protease 2 [KO:K09633] [EC:3.4.21.122]	Up-regulated by androgenic hormones in prostate cancer cells and down-regulated in androgen-independent prostate cancer tissue	Yes				
	*BCL2*	ENSG00000171791	BCL2 apoptosis regulator [KO:K02161]	Involved in the inhibition of apoptosis	Yes				
	*KLK3*	ENSG00000142515	kallikrein related peptidase 3 [KO:K01351] [EC:3.4.21.77]	A protease (PSA) which is synthesized in the epithelial cells of the prostate gland		Yes			
	*NKX3-1*	ENSG00000167034.9	NK3 homeobox 1 [KO:K09348]	Negative regulator of epithelial cell growth in prostate tissue				Yes	
	*AKT2*	ENSG00000105221.16	AKT serine/threonine kinase 2 [KO:K04456] [EC:2.7.11.1]	Protein kinase involved in signalling pathways as oncogene				Yes	
	*CREBBP*	ENSG00000005339.14	CREB binding protein [KO:K04498] [EC:2.3.1.48]	Involved in the transcriptional coactivation of many different transcription factors				Yes	
	*NFKBIA*	ENSG00000100906	NFKB inhibitor alpha [KO:K04734]	Interacts with REL dimers to inhibit NF-kappa-B/REL complexes which are involved in inflammatory responses				Yes	
	*MAPK3*	ENSG00000102882.11	mitogen-activated protein kinase 3 [KO:K04371] [EC:2.7.11.24]	Regulates cell proliferation, differentiation, and cell cycle progression in response to a variety of extracellular signals				Yes	
5′-tRNA-Glu-TTC-9-1_L30	*E2F3*	ENSG00000112242	E2F transcription factor 3 [KO:K06620]	Regulate the expression of genes involved in the cell cycle	Yes	Yes			
	*PTEN*	ENSG00000171862	phosphatase and tensin homolog [KO:K01110] [EC:3.1.3.16 3.1.3.48 3.1.3.67]	Negatively regulates intracellular levels of phosphatidylinositol-3,4,5-trisphosphate in cells and functions as a tumor suppressor by negatively regulating AKT/PKB signalling pathway	Yes	Yes			Yes
	*NRAS*	ENSG00000213281	NRAS proto-oncogene, GTPase [KO:K07828]	Membrane protein with GTPase activity. Oncogene	Yes	Yes	Yes		
	*CREB5*	ENSG00000146592	cAMP responsive element binding protein 5 [KO:K09047]	Specifically binds to CRE as a homodimer or a heterodimer with c-Jun or CRE-BP1, and functions as a CRE-dependent trans-activator	Yes				Yes
	*CREB1*	ENSG00000118260	cAMP responsive element binding protein 1 [KO:K05870]	Transcription factor that induces transcription of genes in response to hormonal stimulation of the cAMP pathway		Yes			Yes
	*CASP9*	ENSG00000132906.17	caspase 9 [KO:K04399] [EC:3.4.22.62]	Plays a central role in apoptosis. Tumor suppressor			Yes		
	*PIK3CA*	ENSG00000121879	phosphatidylinositol-4,5-bisphosphate 3-kinase catalytic subunit alpha [KO:K00922] [EC:2.7.1.153]	Catalytic subunit of PIK3. Oncogenic gene					Yes
	*KRAS*	ENSG00000133703	KRAS proto-oncogene, GTPase [KO:K07827]	Member of the small GTPase superfamily. Proto-oncogene					Yes
	*AR*	ENSG00000169083	androgen receptor [KO:K08557]	Steroid-hormone activated transcription factor					Yes
5′-tRNA-Val-CAC-3-1_L30	*PIK3R2*	ENSG00000105647	phosphoinositide-3-kinase regulatory subunit 2 [KO:K02649]	Lipid kinase that phosphorylates phosphatidylinositol and similar compounds, creating second messengers important in growth signalling pathways			Yes		
	*MAPK1*	ENSG00000100030	mitogen-activated protein kinase 1 [KO:K04371] [EC:2.7.11.24]	Regulates cell proliferation, differentiation, transcription regulation and development	Yes				
5′-M-tRNA-Leu-TAG-1-1_L26	*AKT1*	ENSG00000142208	AKT serine/threonine kinase 1 [KO:K04456] [EC:2.7.11.1]	Regulates cell proliferation, survival, metabolism, and angiogenesis				Yes	
	*BAD*	ENSG00000002330	BCL2 associated agonist of cell death [KO:K02158]	Positively regulates cell apoptosis				Yes	
	*CCND1*	ENSG00000110092	cyclin D1 [KO:K04503]	Required for cell cycle G1/S transition. Interact with tumor suppressor protein Rb					
	*RAF1*	ENSG00000132155	Raf-1 proto-oncogene, serine/threonine kinase [KO:K04366] [EC:2.7.11.1]	MAP kinase kinase kinase (MAP3K) involved in the cell division cycle, apoptosis, cell differentiation and cell migration				Yes	
	*CREB3*	ENSG00000107175	cAMP responsive element binding protein 3 [KO:K09048]	Binds to the cAMP-response element and regulates cell proliferation and tumor suppression				Yes	
	*TCF7L2*	ENSG00000148737	transcription factor 7 like 2 [KO:K04491]	Transcription factor involved in the Wnt signalling pathway					Yes

Note. When a target gene is shared between several tRFs, symbols after the 5′tRF will denote the corresponding predictive model (*: 5′-M-tRNA-Gln-TTG-3-3_L30; ^a^: 5′-tRNA-Glu-TTC-9-1_L30; ^$^: 5′-tRNA-Val-CAC-3-1_L30; ^#^: 5′-M-tRNA-Leu-TAG-1-1_L26).

## Data Availability

Data is contained within the article and Supplementary Materials.

## Supplementary Materials

The following supporting information can be downloaded at: https://www.mdpi.com/article/10.3390/ijms251810122/s1.
