# Supplementary material for: Semen sEV tRF-Based Models Increase Non-Invasive Prediction Accuracy of Clinically Significant Prostate Cancer among Patients with Moderately Altered PSA Levels
Source: Int J Mol Sci. 2024 Sep 20;25(18):10122. doi: 10.3390/ijms251810122 (PMC11432266; doi:10.3390/ijms251810122)
Supplement: Supplementary file 1 [file ijms-25-10122-s001.zip › Supplementary Figures_ijms-3163207_mod.pdf]

Supplementary Figure S1

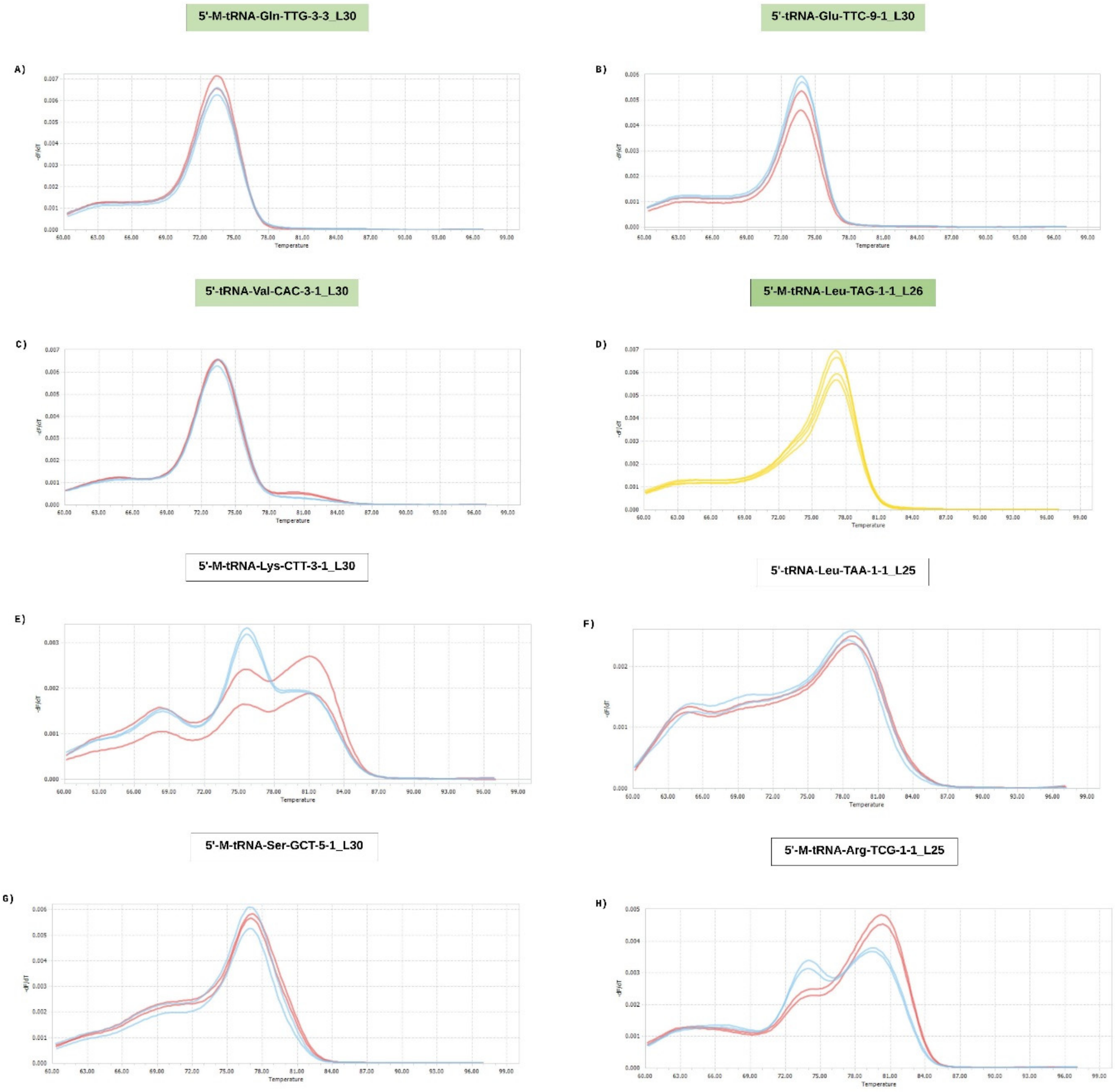

Melting curve analysis of the eight candidate 5'tRFs [(A) 5'-M-tRNA-Gln-TTC-3-3_L30; (B) 5'-tRNA-Glu-TTC-9-1_L30; (C) 5'-tRNA-Val-CAC-3-1_L30; (D) 5'-M-tRNA-TAG-1-1_L26; (E) 5'-M-tRNA-Lys-CTT-3-1_L30; (F) 5'-tRNA-Leu-TAA-1-1_L25; (G) 5'-M-tRNA-Ser-GCT-5-1_L30; (H) 5'-M-tRNA-Arg-TGG-1-1_L25] assayed by miRPrimer 2 RT-qPCR strategy. Only those that presented a single peak (A-D) were further analysed.

Supplementary Figure S2

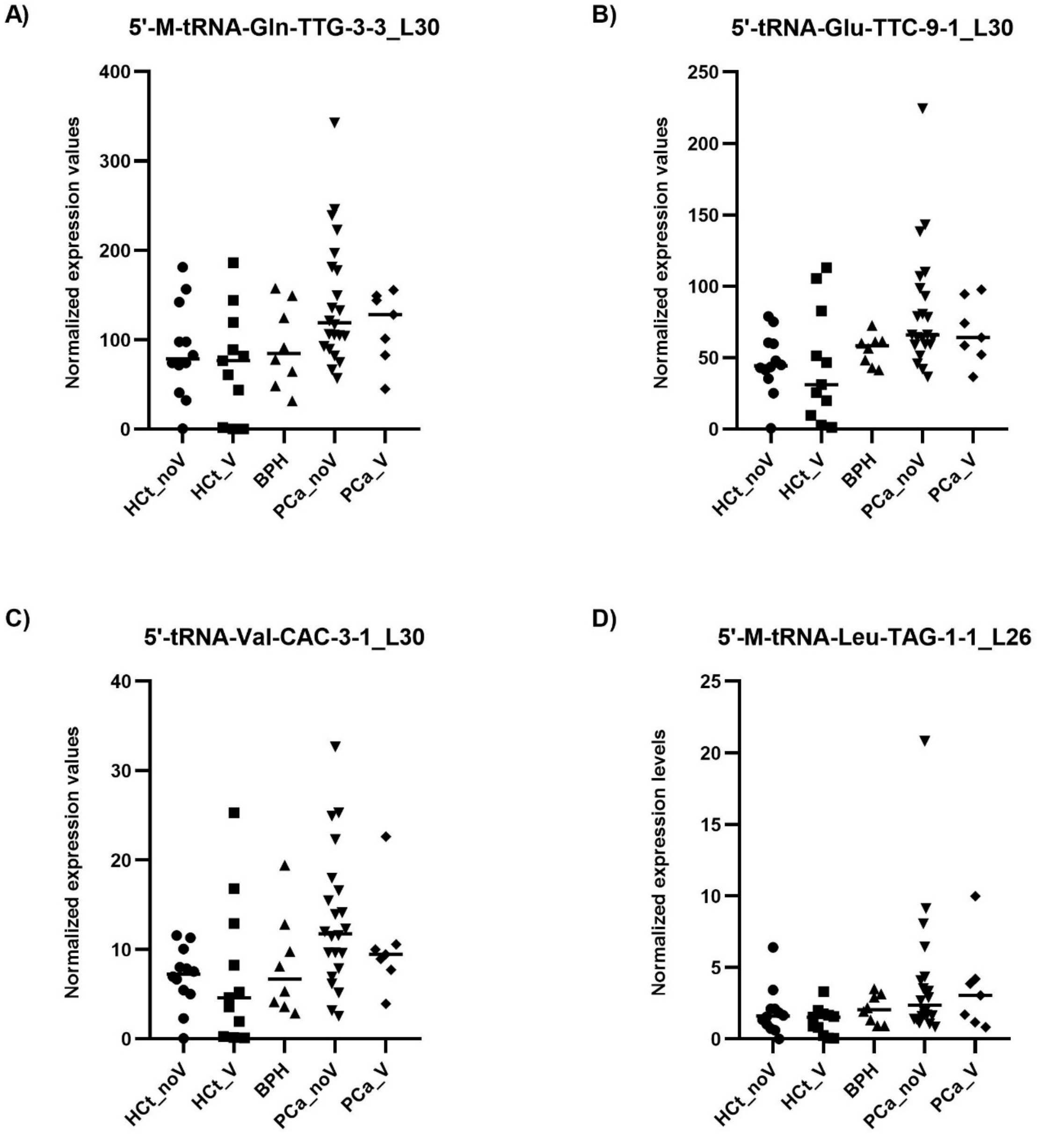

Expression profiling of 5'tRFs [5'-M-tRNA-Gln-TTG-3-3_L30 (A); 5'-tRNA-Glu-TTC-9-1_L30 (B); 5'-tRNA-Val-CAC-3-1_L30 (C) and 5'-M-tRNA-Leu-TAG-1-1_L26 (D)] in EVs from semen. The horizontal bar displays the median expression value. No significant differences between vasectomised (V, square and rhombus respectively) and non-vasectomised (noV, circle and inverted triangle respectively) individuals in both HCt and PCa groups for all tRFs studied was observed (Mann-Whitney U test).

Supplementary Figure S3

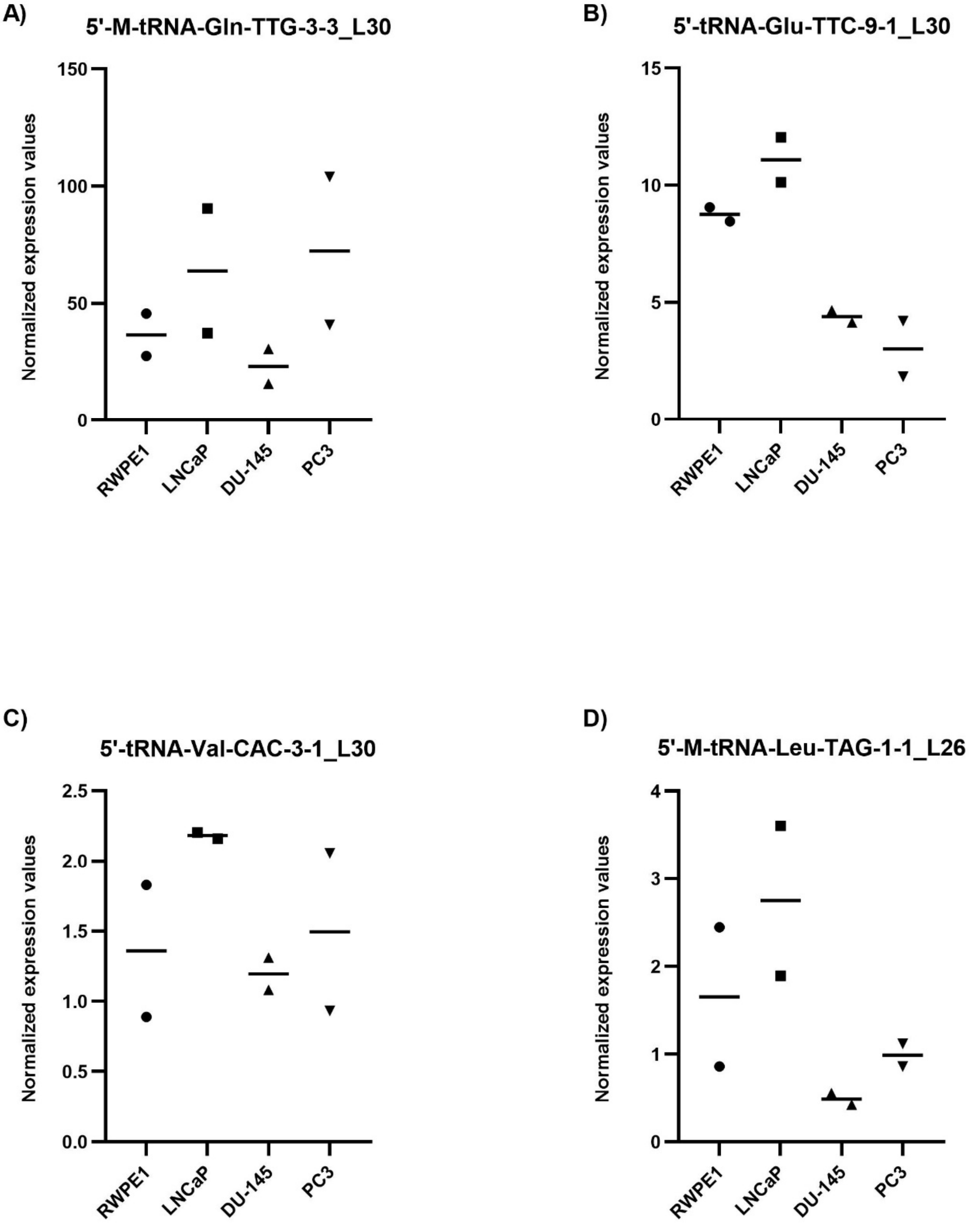

Expression behaviour of selected 5'tRFs in PCa cell lines. 5'-M-tRNA-Gln-TTG-3-3_L30 (A); 5'-tRNA-Glu-TTC-9-1_L30 (B); 5'-tRNA-Val-CAC-3-1_L30 (C) and 5'-M-tRNA-Leu-TAG-1-1_L26 (D) expression was determined by RT-qPCR in human epidermal prostate cells (RWPE1) as control and several PCa cell lines such as androgen sensitive LNCaP and androgen insensitive DU145 and PC3 cell lines. Expression levels relative to miR-30e-3p are shown, which were calculated using the 2dCq strategy.
